# Management of Spontaneous Coronary Dissection Complicated by Cardiogenic Shock: A Case Report

**DOI:** 10.1002/ccr3.72998

**Published:** 2026-07-02

**Authors:** Daniel Grüter, Thomas Seiler, Chiara Schaffner, Adrian Attinger, Florim Cuculi, Matthias Bossard

**Affiliations:** ^1^ Cardiology Division Heart Center‐Luzerner Kantonsspital Luzern Switzerland; ^2^ Department of Internal Medicine Luzerner Kantonsspital Wolhusen Switzerland

**Keywords:** acute coronary syndrome, antiplatelets, cardiogenic shock, out‐of‐hospital cardiac arrest, SCAD

## Abstract

Spontaneous coronary artery dissection (SCAD) is a rare cause of acute coronary syndrome with debated optimal treatment in the setting of cardiogenic shock (CS). We report a case of a middle‐aged woman presenting with out‐of‐hospital cardiac arrest due to extensive multivessel SCAD. Without evidence for transmural ischemia, we chose conservative treatment with single antiplatelet therapy and heparin. Due to deteriorating hemodynamics, a temporary left ventricular assist device (Impella) was implanted, stabilizing the patient. After weaning the device and initiating heart failure therapy, the patient recovered completely. This case highlights several important considerations for the management of SCAD patients with CS.

## Key Clinical Message

In patients with spontaneous coronary artery dissection complicated by cardiogenic shock conservative management supported by temporary mechanical circulatory assistance and left ventricular unloading can be a safe and effective strategy, leading to full recovery when transmural ischemia is absent.

## Graphical abstract

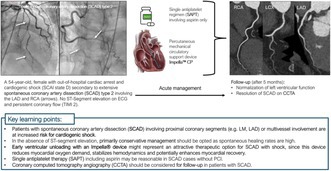

## 1 Introduction

Spontaneous coronary artery dissection (SCAD) is an increasingly recognized cause of acute coronary syndrome, particularly among younger women without traditional cardiovascular risk factors. While the majority of SCAD cases are managed conservatively with favorable outcomes, a subset of patients present with or develop cardiogenic shock [1, 2]. The management of cardiogenic shock in SCAD poses unique challenges, as standard revascularization strategies such as percutaneous coronary intervention (PCI) or coronary artery bypass grafting (CABG) carry a heightened risk of iatrogenic vessel injury in these fragile, dissected arteries. Moreover, limited evidence and a lack of consensus guidelines complicate clinical decision‐making in this setting [3, 4].

Here, we describe a unique case of a female patient with SCAD who developed cardiogenic shock and required mechanical circulatory support, highlighting the complex interplay between SCAD pathology, hemodynamic compromise, and advanced supportive therapies.

## 2 Case History and Examination

A 54‐year‐old, previously healthy woman was admitted to our institution with cardiogenic shock (CS) after out‐of‐hospital cardiac arrest (OHCA). After a down‐time of 10 min, advanced cardiac life support was initiated. The initial rhythm was ventricular fibrillation. Restoration of spontaneous circulation was achieved after 30 min. The patient was intubated in the field. In our emergency department, the patient was in profound cardiogenic shock (SCAI state D) requiring escalating vasopressors (Figure S1).

## 3 Differential Diagnosis, Investigations and Treatment

The initial electrocardiogram (ECG) revealed sinus tachycardia without ST‐segment elevation (STE). The transthoracic echocardiogram (TTE) indicated a depressed left ventricular ejection fraction (LVEF) with wall motion abnormalities in the left anterior descending artery (LAD) territory. Initial pH was 6.9, lactate 11 mmol/L, and high‐sensitivity Troponin T 446 ng/mL. An angio‐computed tomography revealed rib fractures with tension pneumothorax requiring urgent placement of chest drainage.

Our patient remained hemodynamically unstable with clinical signs concerning for ongoing myocardial ischemia, necessitating urgent invasive coronary angiography (ICA). Differential diagnoses at this stage included type 1 myocardial infarction due to coronary thrombus or acute plaque rupture. With respect to the patient's age and sex, we also considered spontaneous coronary artery dissection (SCAD) as a possible diagnosis. Other considerations included coronary vasospasm or embolic phenomena as well as a specific cardiac pathology—for example, cardiomyopathy or channelopathy—leading to ventricular fibrillation. To us, prompt ICA appeared critical to clarify the underlying pathology and guide further management.

**FIGURE 1:**
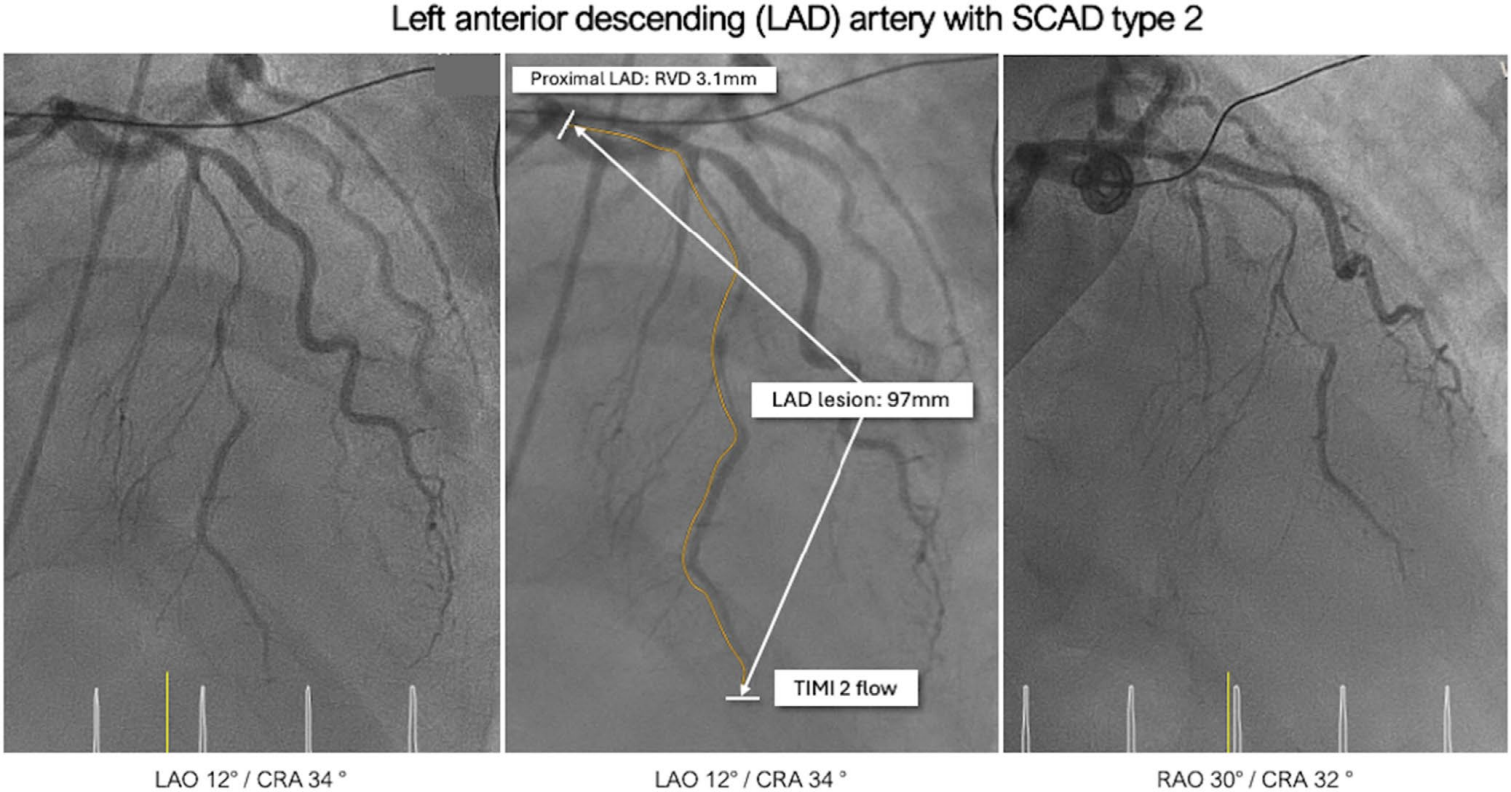
Left anterior descending (LAD) artery with SCAD type 2. Invasive coronary angiography of the left anterior descending (LAD) artery shows extensive spontaneous coronary artery dissection (SCAD) Type 2 from the proximal to the distal segment (SYNTAX epicardial segments 6–8) with persistent coronary TIMI 2 flow (Thrombolysis In Myocardial Infarction Classification). Proximal reference vessel diameter (RVD) 3.1 mm.

**FIGURE 2:**
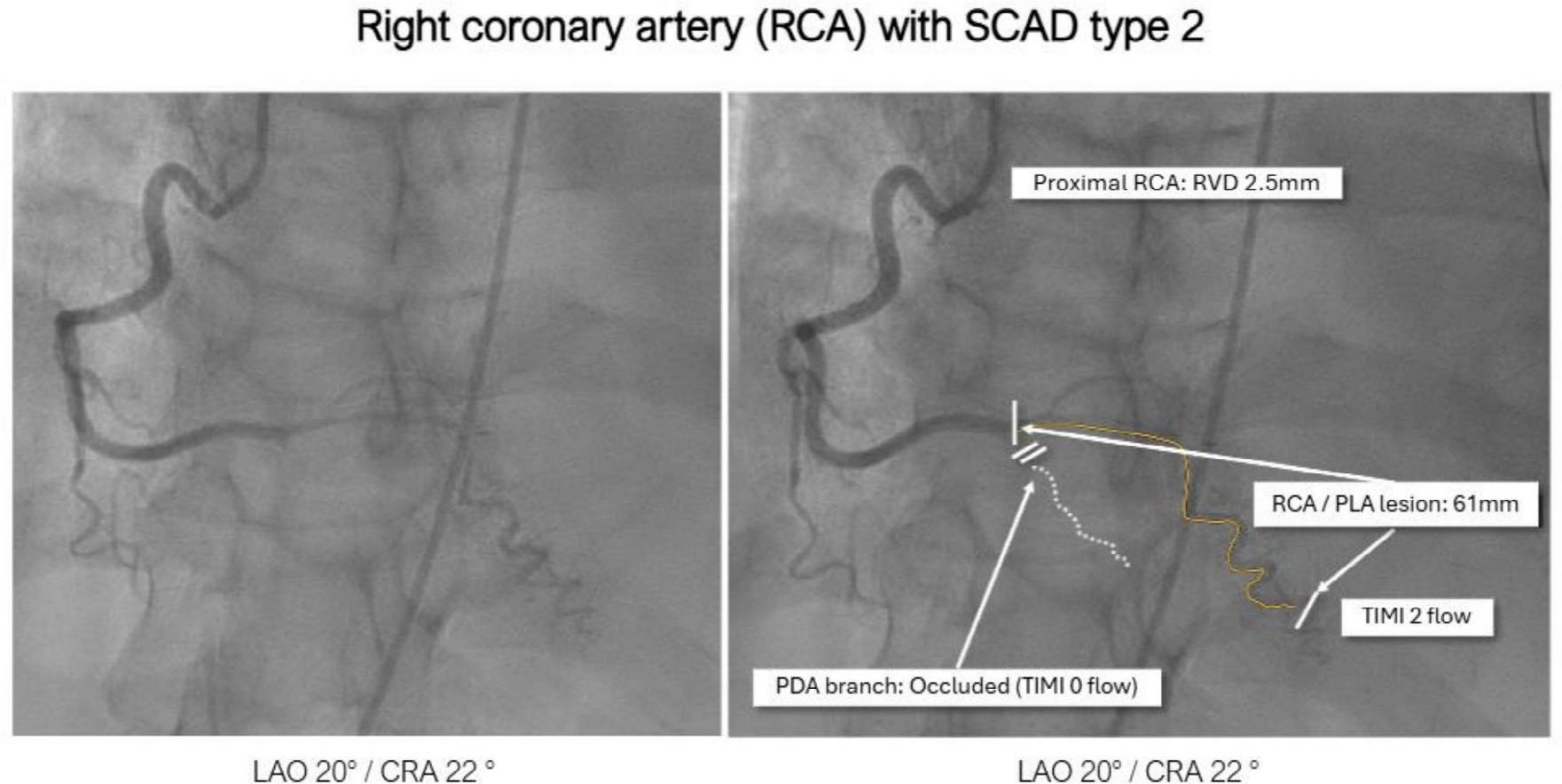
Right coronary artery (RCA) with SCAD type 2. For the right coronary artery (RCA), we found SCAD Type 2 involving the distal RCA—posterolateral branch (PLA) (SYNTAX epicardial segments 3 and 16) with TIMI 2 flow (Thrombolysis In Myocardial Infarction Classification) and the posterior descending artery branch (PDA) (SYNTAX epicardial segment 4) was occluded, TIMI 0 flow. Proximal reference vessel diameter (RVD) 2.5 mm.

**FIGURE 3:**
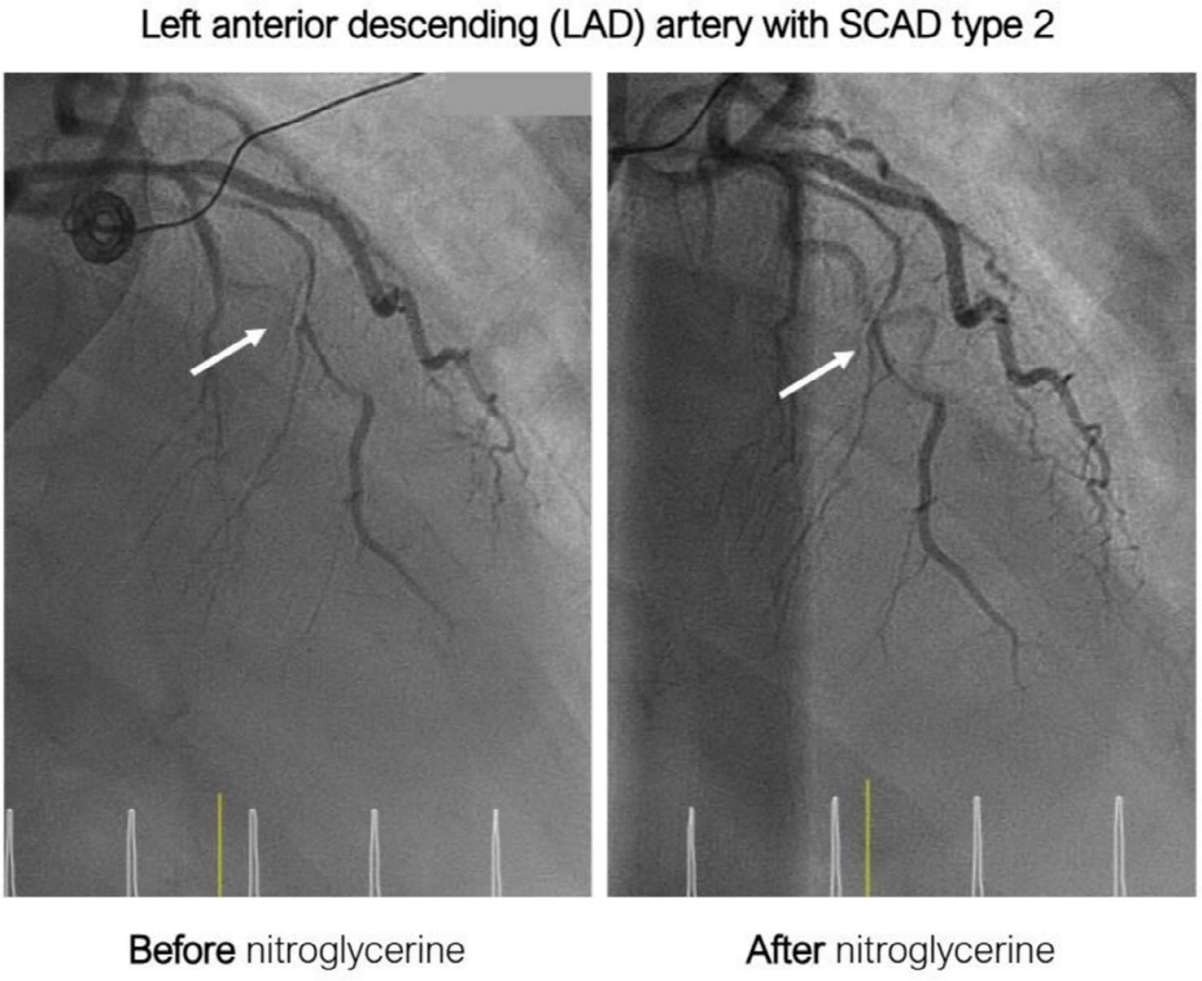
Left anterior descending (LAD) with SCAD Type 2 before and after intracoronary nitroglycerine. Severe coronary vasospasm seemed highly unlikely following the administration of intracoronary nitroglycerine as no change in coronary artery diameter or flow was observed.

ICA revealed extensive dissections involving the LAD and right coronary artery (RCA), consistent with SCAD type 2 (Figures 1 and 2). Given the typical angiographic presentation with multivessel involvement and the concern of false lumen wiring or extension of the dissection or intramural hematoma by catheter manipulation, we limited the angiographic imaging to a minimum and refrained from using intravascular imaging techniques for further assessment. Also, severe coronary vasospasm seemed highly unlikely following the administration of intracoronary nitroglycerin (Figure 3).

Following urgent consultation with the staff cardiac surgeon, we concluded that the extensive dissections involving all major coronary vessels, as well as long and distal segments rendered both PCI and CABG technically unfeasible due to poor distal targets. Additionally, no major coronary artery was completely occluded at the time of angiography and due to the severely reduced LVEF, along with rapidly deteriorating hemodynamics, the risk of revascularization was additionally considered prohibitive in this case. Furthermore, the out‐of‐hospital cardiac arrest (OHCA) with uncertain neurological prognosis led the heart team to ultimately refrain from revascularization.

## 4 Conclusions and Results

**FIGURE 4:**
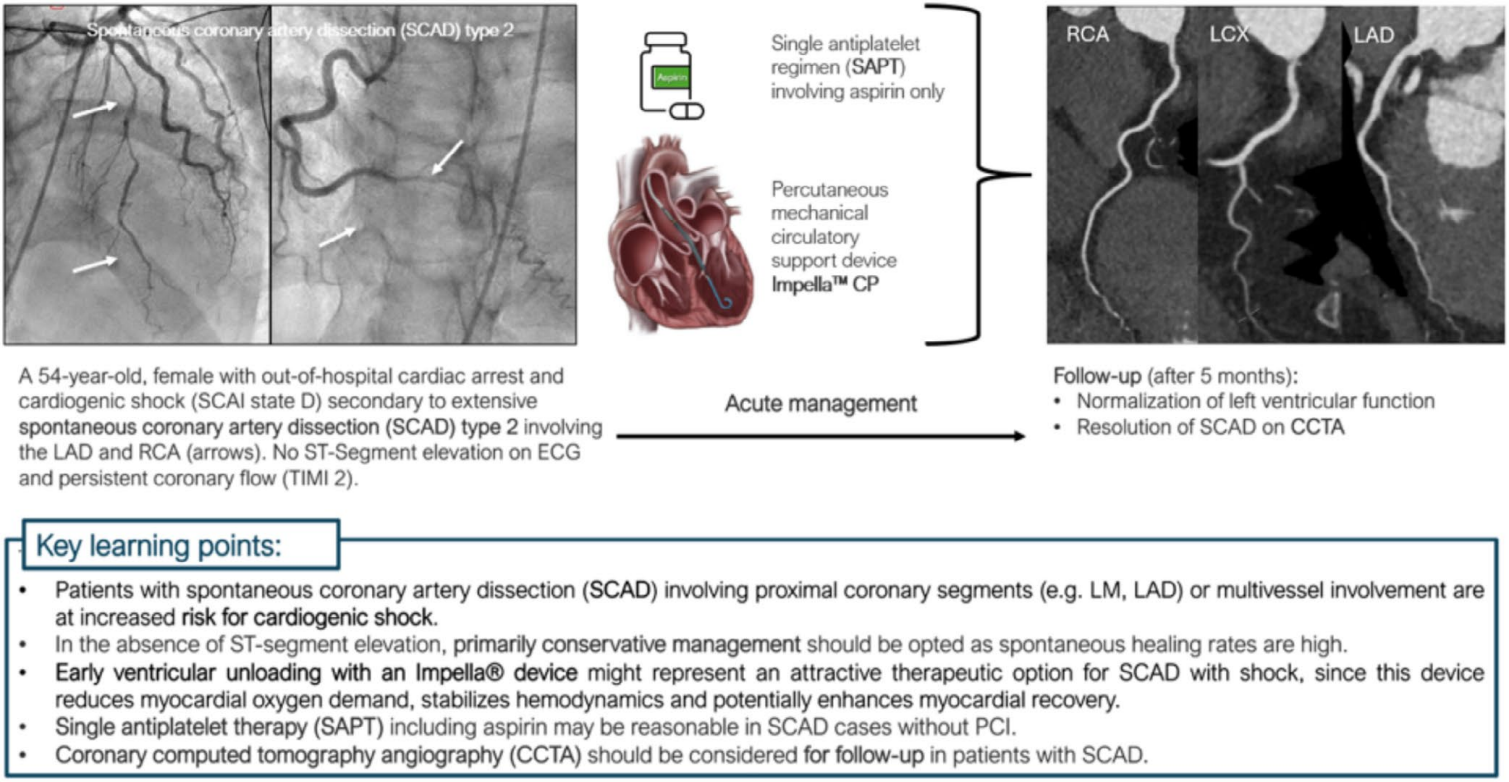
Summary figure. CCTA, Coronary computed tomography angiography; LAD, Left anterior descending coronary artery; LM, Left main coronary artery; PCI, Percutaneous coronary intervention; RCA, Right coronary artery; SAPT, Single antiplatelet therapy; SCAI, Society of Cardiovascular Angiography and Interventions shock classification; SCAD, Spontaneous coronary artery dissection; TIMI, Thrombolysis in myocardial infarction flow classification.

Consequently, our primary focus was on hemodynamic stabilization. A percutaneous left ventricular assist device (pLVAD), Impella CP Smart Assist (Abiomed by J&J, Danvers, Massachusetts), was implanted using a transfemoral approach. Accordingly, intravenous heparinization was initiated in addition to single antiplatelet therapy with aspirin. The patient was transferred to the intensive care unit and underwent 24 h of therapeutic normothermia. After 2.5 days, the pLVAD was successfully weaned, followed by extubation and initiation of heart failure therapy (ACE‐inhibitor, β‐blocker therapy, aldosterone antagonist). The patient was discharged after 17 days and underwent rehabilitation, recovering fully with no clinical residuals and normalized LVEF. Cardiac CT angiography (CCTA) at 5 months follow up showed complete resolution of SCAD (Figure 4).

## 5 Discussion

SCAD remains a rare cause of CS, occurring in only 2%–10% of cases [1]. Given its distinct pathophysiology, particularly in cases with multivessel involvement and progression to shock, guidelines for medical management and revascularization established for type 1 myocardial infarction may not be directly applicable in this context. This case report highlights some important challenges encountered in SCAD patients presenting with cardiogenic shock and provides practical insights into the use of pLVADs, as well as the applicability of revascularization strategies and antiplatelet regimens in such clinical scenarios, as summarized in the *graphical* *abstract*.

Unlike classical type 1 myocardial infarction (MI), generally caused by atherothrombosis, restoring coronary flow in SCAD can be very challenging and acute vessel reconstruction is often impossible [2, 3, 4, 5]. The variability in angiographic presentation and the increased risk of complications make revascularization particularly challenging in SCAD, requiring physicians to carefully balance the risks and benefits of any revascularization strategy. PCI‐related complications like false lumen entry, hematoma propagation and stent underexpansion are common. The outcomes of surgical revascularization depend on local surgical expertise. Moreover, CABG in SCAD patients has been associated with high rates of graft occlusion and is therefore generally performed only when PCI is not feasible (e.g., left main occlusion or multivessel involvement) [5]. Given the high spontaneous healing rate (up to 95% within 30 days) and the aforementioned considerations, conservative management should generally be preferred in the absence of transmural ischemia and revascularization is typically recommended only in cases of ongoing transmural ischemia or CS [3, 4]. Interestingly, recent case reports have also described favorable outcomes with conservative management even in selected cases of left main coronary artery SCAD, supporting a non‐interventional approach in carefully chosen patients [6]. In our case, surgical revascularization seemed impossible due to the extensive dissections. Furthermore, the lacking evidence for transmural ischemia by ECG‐criteria further prompted us to withhold from PCI and primarily focus on hemodynamic stabilization by using a pLVAD.

The pLVAD Impella used in our case not only maintains cardiac output and systemic perfusion but also reduces left ventricular end‐diastolic pressure through ventricular unloading, decreasing wall stress, mechanical work, and myocardial oxygen consumption [5, 6]. This mechanism may not only improve survival, but it also seems to limit infarct size, which determines the long‐term outcome of SCAD patients [7]. Impella CP was favored over intra‐aortic balloon pump (IABP), which provides only limited unloading and hemodynamic support, and over veno‐arterial extracorporeal membrane oxygenation (VA‐ECMO), which may increase afterload, exacerbate left ventricular distension, and is more invasive. In addition, the patient had preserved oxygenation and the absence of right ventricular dysfunction [8]. Managing SCAD complicated by cardiogenic shock necessitates an individualized approach, particularly with respect to revascularization options and the use of mechanical circulatory support. Although evidence is limited, published case series suggest that these patients tend to have favorable long‐term prognoses with appropriate management, often with lower mortality rates than those seen in STEMI or cardiogenic shock secondary to atherosclerotic disease [6].

Finally, optimal medical therapy for SCAD remains controversial, particularly regarding single (SAPT) versus dual antiplatelet therapy (DAPT) in patients not undergoing PCI [1]. On one hand, the thrombotic risk is elevated as platelet activation may occur due to shear stress and exposure to sub‐endothelial tissue at the dissection site. However, true intraluminal thrombus is less common in SCAD compared to atherosclerotic MI [4]. On the other hand, intramural hemorrhage may worsen with potent platelet inhibition and could delay vascular healing and hematoma resorption. A recent observational study linked DAPT to a higher risk of major cardiovascular adverse events within 12 months, driven by non‐fatal MI or unplanned PCI [5]. This may support the use of SAPT in conservatively treated SCAD, as applied in our patient. Of note, our patient received a SAPT regimen with aspirin only, in combination with unfractionated heparin aiming for an activated clotting time (ACT) range of 160–180 s for optimal function of the Impella device.

### 5.1 Key Teaching Points

- Patients with spontaneous coronary artery dissection (SCAD) involving proximal coronary segments (e.g., LM, LAD) or multivessel involvement are at increased risk for cardiogenic shock.
- In the absence of ST‐segment elevation, primarily conservative management should be opted as spontaneous healing rates are high.
- Early ventricular unloading with an Impella device might represent an attractive therapeutic option for SCAD with shock, since it reduces myocardial oxygen demand, stabilizes hemodynamics, and potentially enhances myocardial recovery.
- Single antiplatelet therapy (SAPT) including aspirin may be reasonable in SCAD cases without PCI
- Coronary computed tomography angiography (CCTA) should be considered for follow‐up in patients with SCAD, enabling non‐invasive assessment of coronary healing while avoiding repeat intracoronary instrumentation

## Author Contributions

**Daniel Grüter:** data curation, formal analysis, investigation, methodology, project administration, writing – original draft, writing – review and editing. **Thomas Seiler:** data curation, formal analysis, writing – original draft, writing – review and editing. **Chiara Schaffner:** data curation, writing – review and editing. **Adrian Attinger:** data curation, writing – review and editing. **Florim Cuculi:** conceptualization, resources, writing – review and editing. **Matthias Bossard:** conceptualization, investigation, methodology, project administration, supervision, validation, visualization, writing – original draft, writing – review and editing.

## Funding

The authors have nothing to report.

## Ethics Statement

This case report complies with the principles of the Declaration of Helsinki. In addition, this case report has been approved by the local ethics committee (BASEC ID: 2019‐00274).

## Consent

A written informed consent to publish this case report was obtained from the patient, in accordance with the journal's patient consent policy.

## Conflicts of Interest

D.G., T.S., and C.S. report no conflicts of interest. A.A. has received consulting and speaker fees from SIS Medical, Boston Scientific and Abbott Vascular. F.C. has received consulting and speaker fees from SIS Medical, Abiomed and Abbott Vascular. M.B. has received consulting and speaker fees from Abbott Vascular, Abiomed by J&J Medtech, Acrostak, Amgen, Astra Zeneca, Bayer, Boston Scientifc, Cordis, Daichii Sankyo, NovoNordisk, OM Pharma S.A., Shockwave by J&J Medtech, and SIS Medical.

## Supporting information


**Figure S1:** ICU Hemodynamic Trend and Vasoactive Infusion Record.

## Data Availability

The authors have nothing to report.
