# Supplementary material for: Management of Spontaneous Coronary Dissection Complicated by Cardiogenic Shock: A Case Report
Source: Clin Case Rep. 2026 Jul 2;14(7):e72998. doi: 10.1002/ccr3.72998 (PMC13328213; doi:10.1002/ccr3.72998)
Supplement: Supplementary file 1 — Figure S1: ICU Hemodynamic Trend and Vasoactive Infusion Record. [file CCR3-14-e72998-s001.docx]

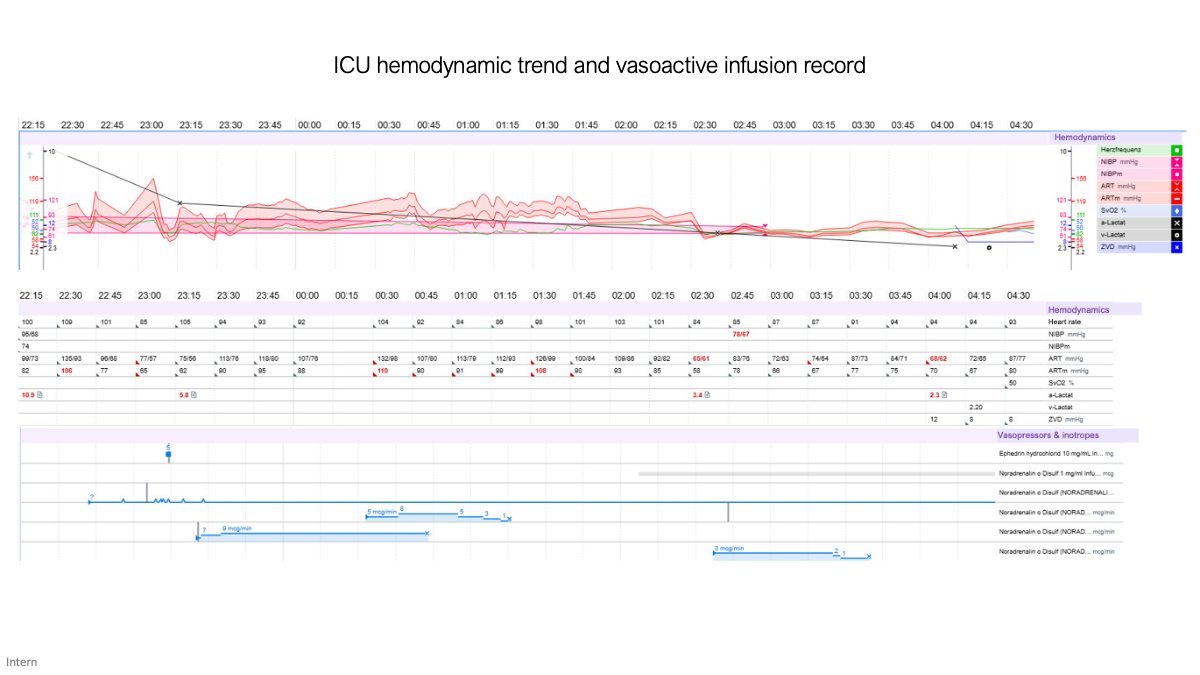


**Supplementary Figure 1 *ICU Hemodynamic Trend and Vasoactive Infusion Record.***ART: arterial blood pressure (invasive, A-line), ICU: intermediate care unit, NBP: non-invasive blood pressure, SvO2: mixed venous oxygen saturation, ZVD: central venous pressure.
